# Torsion of Metastatic Ovarian Tumor Originating From Sigmoid Colon Cancer

**DOI:** 10.7759/cureus.81626

**Published:** 2025-04-02

**Authors:** Maika Uenae, Tomoyuki Sasano, Mari Tomiie, Yoshimi Miyagi, Akihiro Moriyama

**Affiliations:** 1 Department of Obstetrics and Gynecology, Osaka Saiseikai Nakatsu Hospital, Osaka, JPN; 2 Department of Pathology, Osaka Saiseikai Nakatsu Hospital, Osaka, JPN

**Keywords:** bevacizumab, colon cancer, metastasis, ovarian torsion, sigmoid cancer

## Abstract

Metastasis of colorectal cancer (CRC) to the ovaries is relatively rare, and torsion of metastatic ovarian tumors is even rarer. Here, we report the case of a patient with CRC who developed torsion of ovarian metastases and underwent abdominal adnexectomy following chemotherapy with bevacizumab. A 51-year-old premenopausal woman presented with abdominal distension and was referred to our hospital after ultrasonography revealed abdominal tumors. Imaging showed bilateral ovarian masses (maximum diameters: right, 10 cm; left, 14 cm), subserosal leiomyomas, and a circumferential sigmoid colon tumor with peritoneal nodules and ascites. A colonoscopy confirmed moderately differentiated adenocarcinoma. The patient was diagnosed with stage IV sigmoid colon cancer, including ovarian metastasis, peritoneal metastases, and pleural effusion. The patient began chemotherapy with tegafur, gimeracil, oteracil potassium, oxaliplatin, and bevacizumab, resulting in a decrease in carbohydrate antigen 19-9 levels, though carcinoembryonic antigen levels increased. After the fourth chemotherapy cycle, the patient experienced sudden left-sided abdominal pain accompanied by nausea. Computed tomography revealed torsion of the left ovarian tumor (maximum diameter: 19 cm) with suspected hemorrhage. A drop in hemoglobin levels required a blood transfusion. Emergency laparotomy revealed a three-turn torsion of the left ovarian pedicle and an intra-tumoral hemorrhage. A bilateral adnexectomy was performed, and ovarian metastases from colon cancer were confirmed pathologically. Despite bevacizumab treatment, the patient’s postoperative course was uneventful, and she was discharged on day 12. Chemotherapy was resumed. In patients with metastatic ovarian tumors who present with sudden abdominal pain, ovarian torsion should be considered in the differential diagnosis, even in the context of malignancy.

## Introduction

Ovarian torsion is a gynecological surgical emergency caused by the twisting of the ligaments supporting the adnexa (ovary and fallopian tube), which impedes blood flow to varying degrees. Patients typically present with acute-onset moderate-to-severe abdominal or pelvic pain, often accompanied by nausea. Ovarian tumors larger than 5-6 cm in diameter are at increased risk of torsion [1], although ovarian torsion can occur with tumors of any size (mean diameter 9.5 cm, range 1-30 cm) [1]. Ovarian torsion is less common in malignant tumors compared to benign tumors, with the incidence reported at 1.1% for primary and metastatic malignant ovarian tumors, compared to 14.3% for benign tumors [2].

Colorectal cancer (CRC) is the second most commonly diagnosed malignancy and the third leading cause of cancer-related mortality among women worldwide [3]. In Japan, CRC is the leading cause of cancer-related mortality in women, with an incidence rate second only to breast cancer, and this trend continues to rise [4]. While CRC frequently metastasizes to the liver and lungs [5], ovarian metastasis is relatively rare, occurring in approximately 1%-8% of cases (synchronous or metachronous) [6-8]. In ovarian metastases originating from CRC, bilateral involvement is seen in 40% of cases [8,9], with a higher incidence in left-sided, particularly sigmoid, colon cancers [8]. Tumor sizes can reach up to 30 cm [8]. The incidence of torsion in metastatic ovarian tumors originating from CRC is extremely low, likely due to carcinomatous adhesions or peritoneal dissemination, which occurs in 40%-47% of ovarian metastasis cases [5,10,11].

This report describes a patient with sigmoid colon cancer who developed a torsion of metastatic ovarian tumors and underwent abdominal adnexectomy while receiving chemotherapy with bevacizumab.

## Case presentation

**Figure 1:**
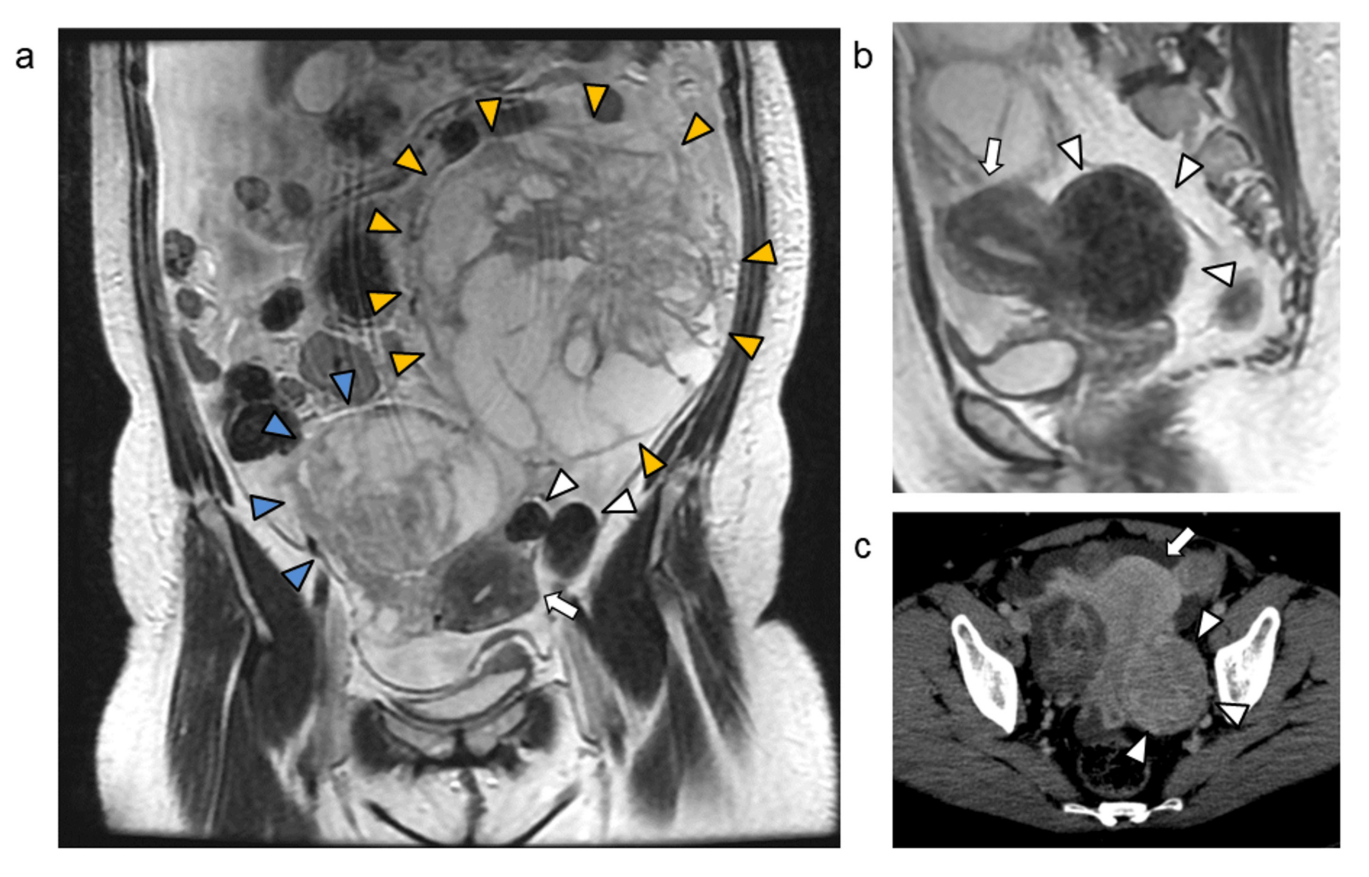
MRI and CT findings at initial diagnosis (a) Abdominal contrast-enhanced coronal T2-weighted MRI showing large masses with multilocular cystic components and mixed solid areas in both adnexa (blue arrowheads, right adnexa; orange arrowheads, left adnexa). Two subserosal leiomyomas (2 and 5 cm) (white arrowheads) are visible on the left side of the uterine fundus (white arrow, uterus). A 7-cm subserosal leiomyoma extending from the lower part of the uterine body to the cervix is depicted on sagittal T2-weighted MRI (b) and axial contrast-enhanced CT (c) (white arrow: uterus).

A 51-year-old premenopausal woman presented with abdominal distension and was referred to our hospital after ultrasonography revealed abdominal tumors. Her medical history included laparoscopic enucleation of a right ovarian endometriotic cyst at age 39. Contrast-enhanced magnetic resonance imaging (MRI) revealed bilateral ovarian tumors, with maximum diameters of 10 cm on the right and 14 cm on the left (Figure 1a). The right ovarian tumor was located within the pelvic cavity, while the left ovarian tumor was positioned superior to the right ovarian tumor. Additionally, subserosal leiomyomas were identified: two on the left side of the uterine fundus, measuring 2 cm and 5 cm in diameter (Figure 1a), and a 7-cm subserosal leiomyoma extending from the lower uterine body to the upper cervix (Figure 1b, 1c).

**Figure 2:**
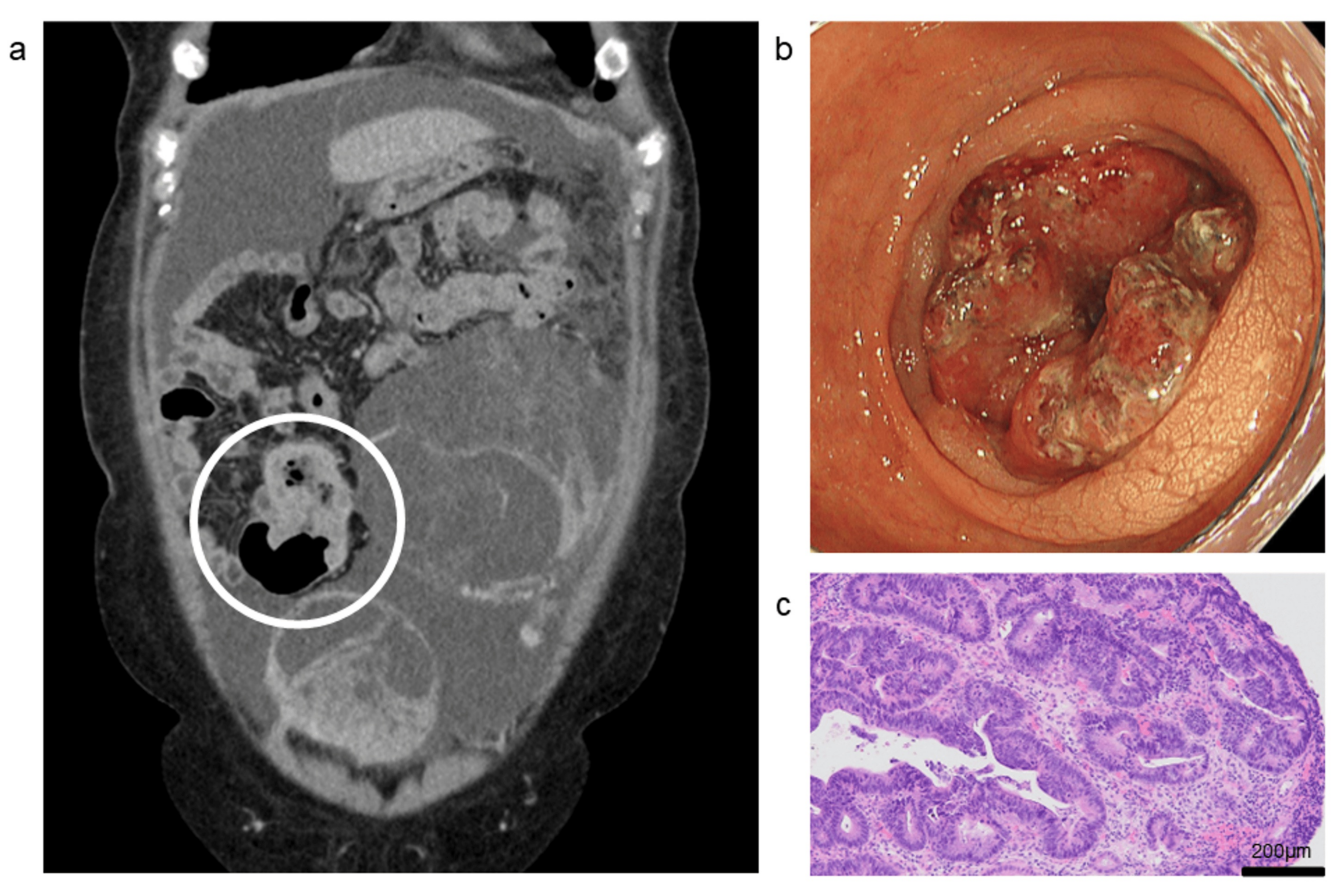
Sigmoid colon cancer (a) Contrast-enhanced abdominal CT showing circumferential thickening of the sigmoid colon wall (circle). (b) Colonoscopy revealing a circumferential tumor measuring 5 cm in length in the sigmoid colon. (c) Hematoxylin and eosin-stained section of colonic mucosal biopsies showing moderately differentiated adenocarcinoma.

Contrast-enhanced computed tomography (CT) revealed circumferential thickening of the tumor in the sigmoid colon (Figure 2a), peritoneal nodules around the left ovarian tumor, a large volume of abdominal ascites, and predominantly right-sided bilateral pleural effusion. Colonoscopy identified a type 2 circumferential sigmoid tumor, located approximately 23 cm from the anal verge and measuring 5 cm in length (Figure 2b). Biopsy confirmed moderately differentiated adenocarcinoma (Figure 2c).

The patient was diagnosed with stage IVC (cT3N0M1c) sigmoid colon cancer, according to the 8th edition of the Union for International Cancer Control (UICC) staging system, with bilateral ovarian metastases and peritoneal metastases, harboring a KRAS p.G12V mutation. The patient underwent chemotherapy with a regimen including tegafur, gimeracil, oteracil potassium (TS-1) (80 mg/m²), oxaliplatin (130 mg/m²), and bevacizumab (7.5 mg/kg). After three cycles, the tumor marker carbohydrate antigen 19-9 (CA19-9) decreased from 83.2 U/mL to 49.1 U/mL (normal rage <37 U/mL), whereas carcinoembryonic antigen (CEA) increased from 5.79 ng/mL to 13.86 ng/mL (normal range <5.00 ng/mL) compared to the levels at initial diagnosis.

**Figure 3:**
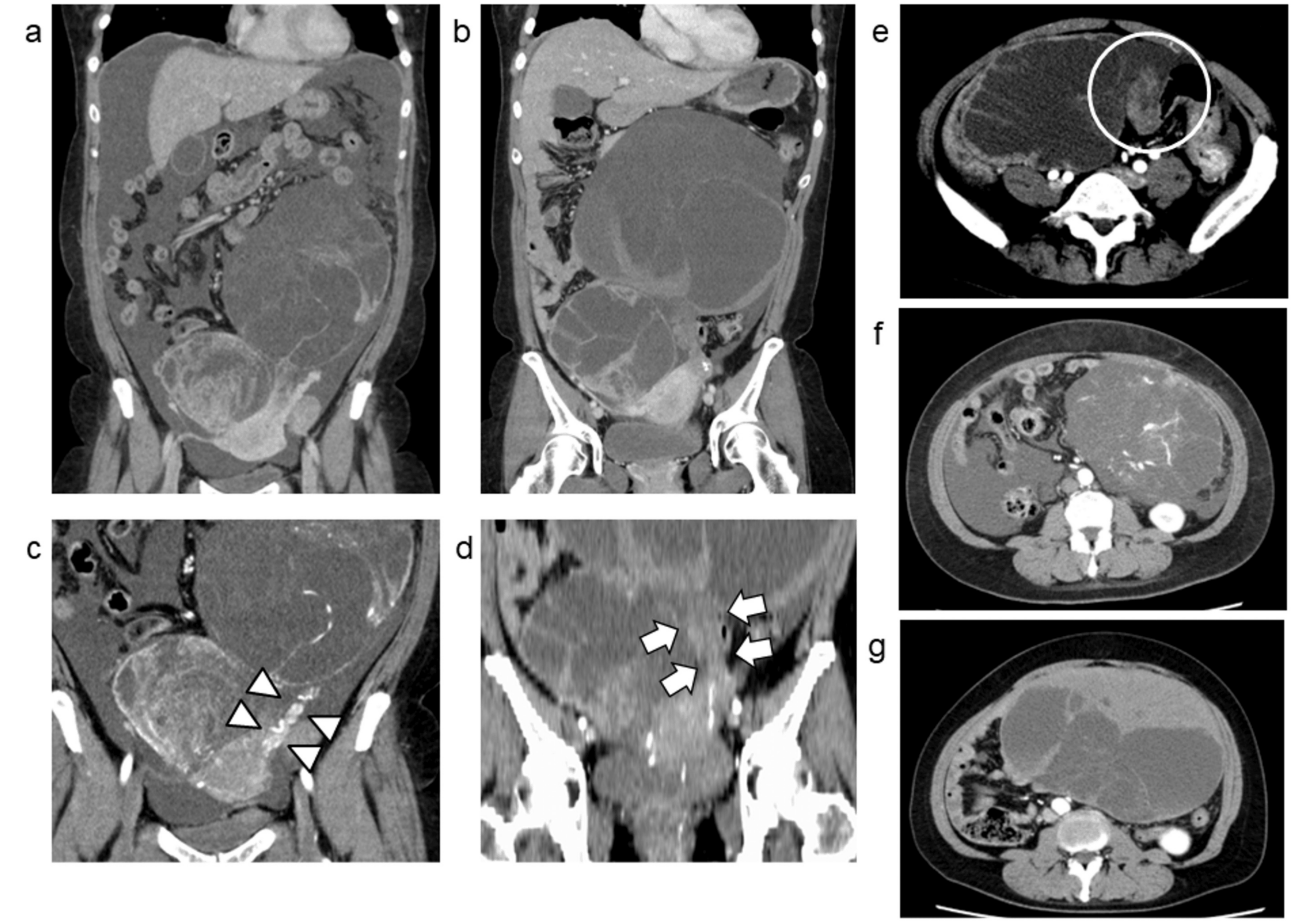
Changes on CT images before and after torsion Coronal (a-d) and axial (e-g) contrast-enhanced CT images in the venous phase (a, b) and arterial phase (c-g). Swollen left and right ovarian tumors are present after torsion (b) compared to before torsion (at initial diagnosis) (a). Contrast enhancement is visible in the artery within the left infundibulopelvic ligament (arrowheads) before torsion (c). After torsion, there is an absence of enhancement in the ovarian and/or infundibulopelvic ligaments (arrows) (d). A twisted pedicle sign is indicated (circle) (e). Contrast enhancement is observed before torsion (f). Following torsion, contrast enhancement is absent, and a relatively high-attenuation area ventral to the tumor suggests hemorrhage or edema (g).

Seven days after the fourth chemotherapy cycle, the patient experienced sudden pain from the left abdomen to the left groin, accompanied by nausea. Her white blood cell count was mildly elevated at 8.1 × 10^3^/µL, but remained within the normal range, and C-reactive protein was stable at 0.07 mg/dL. Hemoglobin level was 12.6 g/dL, with no evidence of anemia. Contrast-enhanced CT revealed that the left and right ovarian tumors had increased to 19 cm and 13 cm, respectively (Figure 3a, 3b). The left ovarian tumor exhibited signs suggestive of torsion (Figure 3c-3g), and intra-tumoral hemorrhage or ovarian edema was suspected (Figure 3g).

On the third day of hospitalization, the patient's hemoglobin level dropped to 8.3 g/dL, necessitating the transfusion of two units of erythrocytes. No imaging findings suggestive of gastrointestinal perforation or ovarian tumor rupture were observed.

**Figure 4:**
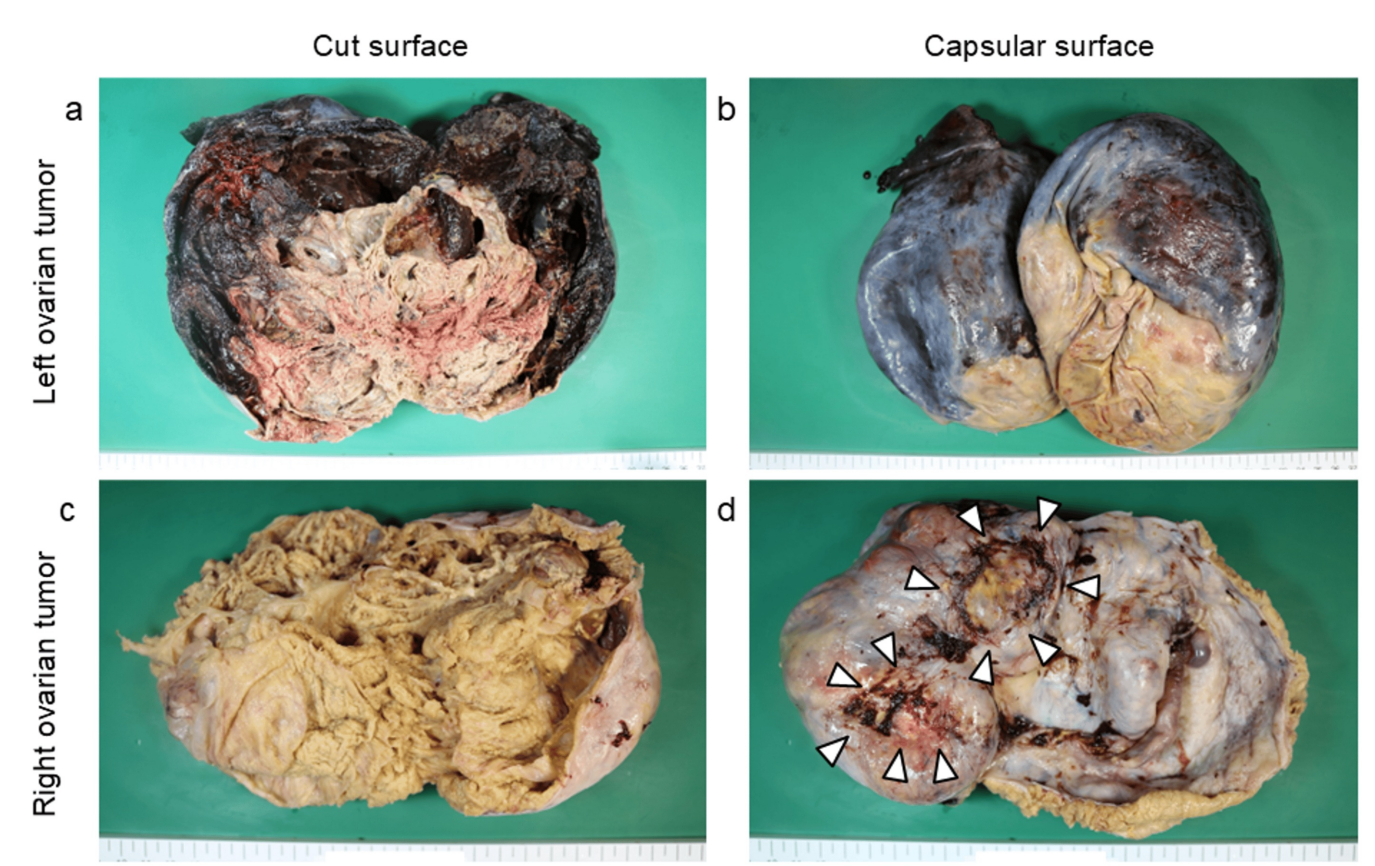
Gross appearance of the left and right ovarian tumors (a, b) Left ovarian tumor with torsion. The cut surface predominantly shows intra-tumoral hemorrhage and necrosis. The capsular surface shows no evidence of peritumoral adhesion. Tumor weight: 1.6 kg. (c, d) Right ovarian tumor without torsion. The cut surface shows no evidence of intra-tumoral hemorrhage, and detached adhesions (arrowheads) are visible on the capsular surface. Tumor weight: 0.82 kg.

After coordination by the gastrointestinal surgical team, an emergent laparotomy was performed. After a midline lower abdominal incision extending 3 cm above the umbilicus, the 13-cm right ovarian tumor, located caudal to the left, was exteriorized following blunt dissection from the surrounding adhesions. Subsequently, the left ovarian tumor was removed, which presented as a dark-red mass extending into the upper abdomen. A three-turn counterclockwise torsion of the left ovarian pedicle was identified. During exteriorization, a partial rupture occurred, releasing a brownish fluid suggestive of intra-tumoral hemorrhage. Left adnexectomy was performed, and after achieving hemostasis with active bleeding from the dissection plane of the right ovarian tumor, bilateral adnexectomy was completed. The operative time was 136 minutes, with a blood loss of 1,003 mL (including the hematoma content). Four units each of erythrocytes and fresh-frozen plasma were transfused intraoperatively. Macroscopic appearance revealed intra-tumoral hemorrhage and necrosis in the left ovary (Figure 4a, 4b), whereas the right ovary showed no hemorrhage (Figure 4c, 4d).

**Figure 5:**
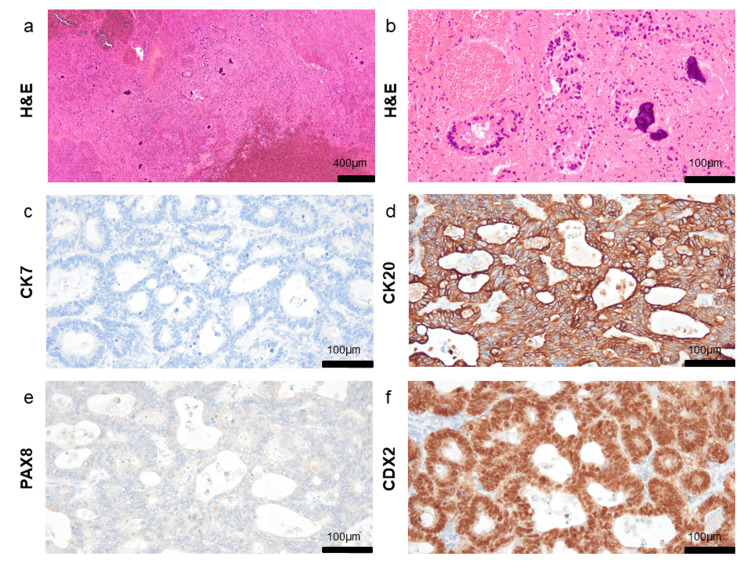
Microscopic pathological examination findings of the left and right ovarian tumors (a, b) Hematoxylin and eosin (H&E) staining of the left ovarian tumor showing hemorrhage and necrotic changes, consistent with moderately differentiated adenocarcinoma, which correlates with the biopsy tissue from the sigmoid colon cancer (b). Immunohistochemical staining of the right ovarian tumor: (c) CK7 negative, (d) CK20 positive, (e) PAX8 negative, (f) CDK2 positive.

Based on the results of hematoxylin and eosin staining and immunohistochemical staining, pathological examination confirmed bilateral ovarian metastases originating from colon cancer (Figure 5). The tumor cells were negative for cytokeratin 7 (CK7) and paired box protein-8 (PAX8), and positive for cytokeratin 20 (CK20) and caudal-type homeobox transcription factor 2 (CDX2) (Figure 5c-f).

Although surgery was performed 11 days after the last dose of bevacizumab, the immediate postoperative course was uneventful, and the patient was discharged on postoperative day 12. However, during the post-discharge period, the patient developed obstructive symptoms, likely due to anastomotic stenosis at the primary tumor site, compounded by impaired postoperative intestinal motility. Ten days after discharge, a laparoscopic diverting transverse colostomy was performed. The patient was discharged on postoperative day 10 following colostomy creation without any complications. Adjuvant chemotherapy was resumed three weeks after colostomy surgery, with bevacizumab reintroduced in the second month post-colostomy. Tumor marker surveillance demonstrated a favorable response, with CA19-9 and CEA levels declining to 9.9 U/mL and 1.91 ng/mL, respectively, one month after surgery for torsion.

## Discussion

In a patient with sigmoid colon cancer and bilateral ovarian metastases, emergency surgery was successfully performed for metastatic ovarian torsion within two weeks of bevacizumab treatment, with no postoperative complications. This case highlights the following key points: (1) Even in patients with metastatic ovarian tumors and peritoneal metastases of CRC who present with stable symptoms during chemotherapy, ovarian tumor torsion should be considered in the differential diagnosis of acute abdominal pain. (2) Despite bevacizumab administration, the patient demonstrated a favorable postoperative course with no complications.

Ovarian torsion in metastatic ovarian tumors from CRC is extremely rare. To the best of our knowledge, only two cases of torsion in metastatic ovarian tumors derived from CRC have been reported [12,13]. Both cases involved unilateral torsion, one in the right ovary and the other in the left, with tumor sizes of 6.6 cm (tumor size unknown in one case). The metastases originated from the rectosigmoid colon (in one case) and from an unknown primary (in the other). In both cases, ovarian torsion due to metastatic CRC was diagnosed only during emergency surgery following the onset of abdominal pain, or through postoperative pathological examination. In our case, unilateral left ovarian torsion (tumor size: 19 cm) occurred during chemotherapy in the context of bilateral ovarian metastases from sigmoid colon cancer, with the right ovarian tumor (tumor size: 13 cm) adhering to the surrounding organs and fixed within the right pelvic cavity. Based on the macroscopic appearance in this case (Figure 4), no adhesions were found around the left ovarian tumor, leading us to conclude that only the left ovary underwent torsion.

We explored potential reasons for the absence of adhesions surrounding the left ovarian tumor. The right ovarian tumor impacted the pelvic cavity and adhered to the surrounding structures, as observed in the operative findings. Additionally, a 7-cm subserosal leiomyoma was present, extending from the lower uterine segment to the cervix, along with two subserosal leiomyomas measuring 2 and 5 cm on the left side of the uterine fundus. These leiomyomas and the fixed right ovarian tumor may have contributed to the narrowing of the Douglas pouch and left pelvic cavity. Consequently, the enlarged left ovarian tumor was displaced superiorly above the impacted right ovarian tumor into the upper abdomen. In the upper abdomen, the left ovarian tumor likely exhibited greater mobility than in the pelvic or lower abdominal regions, owing to increased space and abdominal wall movement. This enhanced mobility may have resulted in reduced adherence. Furthermore, the observed reduction in peritoneal metastatic lesions following treatment initiation may have improved the carcinomatous inflammation in the abdominal cavity, preventing adhesion of the left ovarian tumor.

After three cycles of chemotherapy, the tumor marker levels of CA19-9 decreased, but CEA levels increased. On CT, the tumor size in the sigmoid colon showed no change, and the size of the peritoneal metastases decreased. The ascites almost resolved, although a small volume of right pleural effusion persisted. At the time of the initial diagnosis, malignant pleural effusion was not pathologically confirmed, and no pleural nodules were observed on CT. Considering the disappearance of the right pleural effusion two weeks after the removal of the large bilateral ovarian tumor, pseudo-Meigs syndrome could be a possible explanation [14]. Although the lesions appeared globally stable, except for the ovaries, after the start of chemotherapy, the size of bilateral ovarian tumors alone increased. The cause of this selective increase in ovarian tumor size remains unclear. Sekine et al. reported that ovarian metastases were less responsive to systemic chemotherapy than extra-ovarian metastases in patients with metastatic CRC [15]. Some studies report the rupture of metastatic ovarian tumors originating from CRC [16,17], possibly due to the rapid growth of the tumor [17]. We must be vigilant for complications such as rupture, bleeding [18], and torsion in patients with ovarian metastasis of CRC. Additionally, even in the absence of marked changes in subjective symptoms during chemotherapy, we must be aware that enlarged metastatic ovarian tumors can cause torsion in patients with CRC.

Bevacizumab is frequently used in combination with chemotherapy in patients with CRC. In this case, intra-tumoral hemorrhage of the left ovarian tumor was likely the consequence of ovarian torsion, but bevacizumab administration may have exacerbated bleeding in the tumor. The decision to perform surgery during bevacizumab treatment may be challenging for clinicians. Because bevacizumab has a half-life of 17-21 days, it is recommended to wait 4-6 weeks before surgery to mitigate risks, such as delayed wound healing, gastrointestinal perforation, and thromboembolism [19]. In this case, the patient received bevacizumab treatment, and surgery was performed on the 11th day after the final dose. Fortunately, the patient's postoperative course following the surgery for torsion was uneventful, with no complications related to bevacizumab. Similarly, a case report described a successful bilateral adnexectomy performed 16 days after the final bevacizumab dose in a patient undergoing CRC treatment for ovarian torsion caused by a primary ovarian tumor, without severe complications [20]. Since a twisted ovarian tumor is expected to be non-adherent to the surrounding tissues, complete resection is believed to be achievable if the ovarian tumor and its ligaments can be accessed after an abdominal wall incision. It is important for gastroenterologists, gastroenterological surgeons, and gynecologists to carefully discuss the timing and methods of surgery.

## Conclusions

We encountered a case of colon cancer with ovarian tumor torsion, in which adnexectomy was successfully performed without any complications during bevacizumab administration. In patients with metastatic ovarian tumors who present with sudden abdominal pain, torsion should be considered in the differential diagnosis, even in the context of malignancy.
